# Reliable and Rapid Detection and Quantification of Enrofloxacin Using a Ratiometric SERS Aptasensor

**DOI:** 10.3390/molecules27248764

**Published:** 2022-12-10

**Authors:** Panxue Wang, Li Wang, Cen Li, Xiang Li, Guoliang Li

**Affiliations:** School of Food Science and Engineering, Shaanxi University of Science & Technology, Xi’an 710021, China

**Keywords:** enrofloxacin, detection, SERS aptasensor, ratiometric, portable Raman spectrometer, food safety

## Abstract

Reliable detection and quantification of antibiotic residues in food using surface-enhanced Raman spectroscopy remain challenging, since the intensities of SERS signals are vulnerable to matrix and experimental factors. In this work, a ratiometric SERS aptasensor using 6-Carboxyl-X-Rhodamine (ROX)-labeled aptamers and 4-mercaptobenzonitrile (4-MBN)-functionalized gold nanoparticles (Au NPs) as SERS probes was established for the reliable and rapid detection and quantification of enrofloxacin. In the presence of enrofloxacin, the conformational transform of aptamers took place, and the distance between ROX and Au NP increased, which resulted in a decrease in the SERS signal intensity of ROX. Meanwhile, the intensity of the SERS signal of 4-MBN was used as an internal standard. Reliable determination of enrofloxacin was realized using the ratio of the SERS signal intensities of ROX to 4-MBN. Under optimal conditions, the developed ratiometric SERS aptasensor provided a wide linear range from 5 nM to 1 µM, with a correlation coefficient (R^2^) of 0.98 and a limit of detection (LOD) of 0.12 nM (0.043 ppb). In addition, the developed ratiometric SERS aptasensor was successfully applied for the determination of enrofloxacin in fish and chicken meat, with recovery values of 93.6–112.0%. Therefore, the established ratiometric SERS aptasensor is sensitive, reliable, time-efficient, and has the potential to be applied in the on-site detection of enrofloxacin in complex matrices.

## 1. Introduction

Antibiotics have been extensively used in recent years in human and veterinary medicine to treat various diseases [1]. Among them, fluoroquinolone antibiotics, which exhibit a broad spectrum of antibacterial activities, have been widely applied in the treatment of infectious diseases in humans and animals, including gastrointestinal, respiratory, and urinary tract bacterial infections [2,3]. In addition, fluoroquinolone antibiotics are widely used in aquaculture production to inhibit the microbial propagation and to prevent the rot of aquatic products [4,5]. Enrofloxacin is a fluoroquinolone antimicrobial drug, and has been widely employed in animal husbandry in order to treat diseases or improve growth and productivity [6]. However, the overuse and abuse of antibiotics leads to the broad occurrence of antibiotic resistance in bacteria [7]. Antibiotic residues in animal-derived food pose a serious threat to human health. Therefore, different countries have established maximum residue limits (MRLs) of antibiotics in animal-derived food [8,9]. For enrofloxacin, the MRLs range from 100 µg/kg to 300 µg/kg, depending on the animal species and the target tissue [10]. Reliable and rapid detection of antibiotic residues in animal-derived food is of great importance for monitoring antibiotic residues in animal-derived food and for ensuring food safety.

Currently, various analytical techniques, including high-performance liquid chromatography (HPLC) [11], HPLC coupled with ultraviolet detect (HPLC-UV) and fluorescence detection (HPLC-FLD) [12], liquid chromatography combined with mass spectrometry (LC-MS) [13,14], microbial methods [9], enzyme-linked immunosorbent assay (ELISA) [15], immunochromatographic test strip [16], and the molecular imprinting technique [17] have been developed for the detection of enrofloxacin residues. These methods possess good sensitivity and accuracy, but are time consuming, expensive, and require large-scale instruments, tedious operating procedures, and skilled personnel, greatly limiting their application in rapid detection and on-site detection [18,19]. Therefore, researchers have been making efforts to develop facile, rapid, and reliable detection methods for the determination of antibiotic residues, such as fluorescence spectroscopy methods [20], colorimetric methods [21], electrochemical biosensors [22] and surface-enhanced Raman scattering (SERS)-based methods [23]. In particular, SERS has attracted extensive attention due to its advantages, which include ease of operation, rapid response, single-molecule-level sensitivity, and fingerprint identification capacity [24].

SERS is an ultrasensitive vibrational spectroscopic technique that combines Raman scattering and nanotechnology [25,26]. To date, many efforts have been made to develop SERS strategies for the detection of enrofloxacin, mainly focusing on the preparation of novel SERS-active substrates. For instance, SiO_2_ membranes embedded with silver nanoparticles (Ag NPs) were successfully synthesized by Tang et al. and applied to the detection of enrofloxacin [27]. Chen et al. developed flexible and cost-effective Ag/Nanocellulose fibers and used them for the in situ detection of enrofloxacin [28]. Additionally, monolayer Ag film prepared by inducing self-assembly of Ag NPs on the interface of water/oil was applied for the sensitive and reproducible SERS detection of enrofloxacin [29]. Nevertheless, these substrates not only involve complex preparation processes, they also have an uneven distribution of hot spots, and are difficult to apply in quantitative determination. Therefore, a ratiometric SERS aptasensor for the reliable and rapid detection and quantification of enrofloxacin in animal-derived food was designed in this study.

Aptamer is an artificial single-stranded DNA or RNA oligonucleotides with a length of 10–100 bases, and is selected by the systematic evolution of ligands using an exponential enrichment (SELEX) process [30]. Aptamers can specifically and efficiently bind with targets by folding into specific spatial conformations. Considering their advantages of low cost, high affinity and specificity, easy modifiability, and long-term storage, aptamers have been used extensively in bioanalysis [25], medicine [31] and food safety analysis [32]. In particular, the aptamer-based SERS sensing strategy, which effectively combines the high sensitivity of SERS with the excellent affinity of aptamers, has attracted great attention for the determination of antibiotic residues [33]. However, SERS aptasensors with reliable detection and quantification capacity are still needed, due to the frequently occurring fluctuations in the intensities of SERS signals and the poor reproducibility of SERS analysis [34]. Considering this, a ratiometric SERS aptasensor is proposed in this study to surmount the fluctuations of SERS signals and the effects of matrix and experimental factors [33]. Ratiometric SERS analysis makes use of two different Raman labels, with one of them being used to respond to the target and the other being used as an internal standard to eliminate interference [35].

In this study, a ratiometric SERS aptasensor for the detection of enrofloxacin was established using 6-Carboxyl-X-Rhodamine (ROX)-labeled aptamers and 4-mercaptobenzonitrile (MBN)-functionalized Au NPs as the SERS probe. In the presence of enrofloxacin, the conformation of the aptamers changes due to the specific recognition of enrofloxacin by the aptamers, leading to a corresponding increase in the distance between ROX and Au NP and a decrease in the intensities of the SERS signals of ROX. Meanwhile, the intensities of the SERS signals of 4-MBN are used as internal standards to eliminate interference. The ratio of the SERS signal intensities of ROX to 4-MBN is used to achieve accurate and sensitive determination and quantification of enrofloxacin. To the best of our knowledge, this is the first time a ratiometric SERS aptasensor has been established for the reliable and rapid detection and quantification of antibiotic residues. The developed ratiometric SERS aptasensor has the advantages of being easy to operate, rapid to respond, and reliable for both determination and quantification in comparison with previously reported SERS methods for enrofloxacin detection. Therefore, the developed ratiometric SERS aptasensor provides a new strategy for the determination and quantification of antibiotic residues in complex matrices and has the potential to be applied as an on-site detection technique.

## 2. Results and Discussion

### 2.1. Mechanism of the Ratiometric SERS Detection of Enrofloxacin

**Scheme 1:**
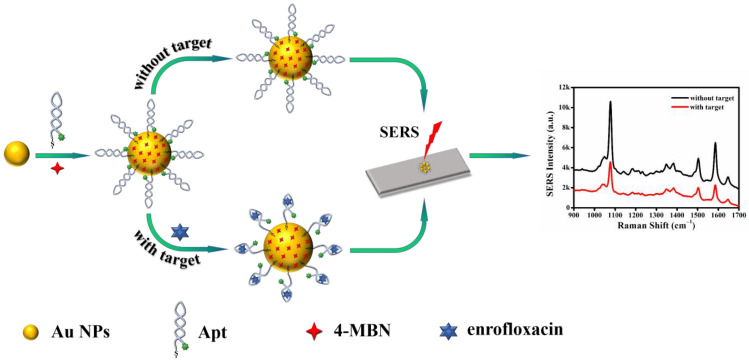
Schematic diagram of enrofloxacin detection using the developed ratiometric SERS aptasensor.

The principle of the developed ratiometric SERS aptasensor for enrofloxacin detection is illustrated in Scheme 1. The aptamer used in this study was modified with HS-SH-(CH_2_)_6_ at the 5′ end and labeled with ROX at the 3′ end. In addition, the sequence of the aptamer was artfully designed by adding five bases at the 5′ end and two bases at the 3′ end of a reported truncated aptamer [21] in order to achieve an optimal distance between ROX and Au NPs and to achieve a superior SERS signal of ROX. The secondary structure of the aptamer was predicted by NUPACK [36] and is given in Figure S1. The SERS probes were synthesized by modifying the designed aptamers and 4-MBN on the surface of Au NPs via Au-S bond. As displayed in Scheme 1, in the absence of enrofloxacin, a stronger SERS signal of ROX and 4-MBN was collected. In addition, in the presence of enrofloxacin, aptamers on the surface of Au NPs would specifically bind with enrofloxacin, resulting in the conformation transform of aptamers and an increase in the distance between ROX and the Au NPs. Therefore, the intensities of the SERS signals of ROX decreased after the binding of aptamers with enrofloxacin, since the effect of SERS enhancement is related to the distance between the target and the enhancing substrate. Meanwhile, the SERS signals of 4-MBN can be used as internal standards to calibrate the intensities of the SERS signals of ROX. Therefore, reliable detection and quantification of enrofloxacin can be achieved on the basis of the ratio of the SERS signal intensities of ROX to 4-MBN using the established SERS aptasensor.

### 2.2. Characterization of the SERS Probe

**Figure 1:**
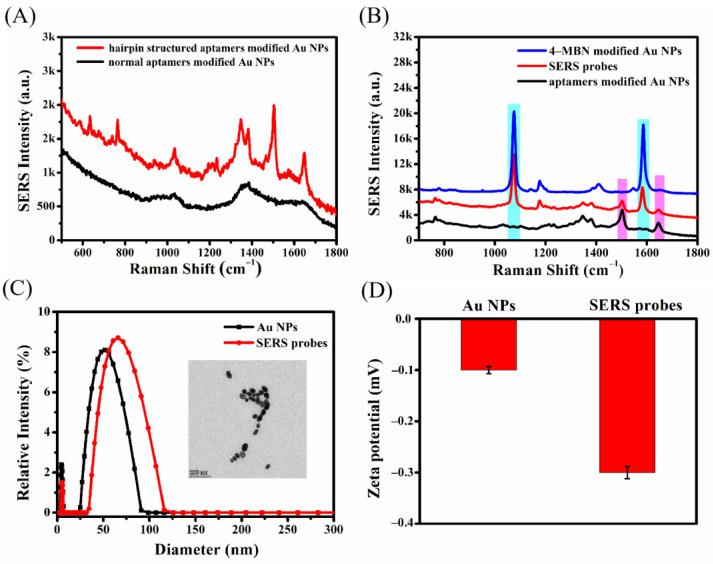
Results of the characterization of the SERS probe. (**A**) SERS spectra of normal aptamer- and hairpin-structured aptamer-modified Au NPs. (**B**) SERS spectra of 4-MBN-modified Au NPs, SERS probes, and hairpin-structured aptamer-modified Au NPs. (**C**) Size distribution of Au NPs and SERS probes (main panel) and a representative TEM image of Au NPs (inset panel). (**D**) Zeta potentials of Au NPs and SERS probes.

The SERS probes used in this study were prepared by conjugating hairpin-structured aptamers and 4-MBN on Au NPs. SERS, dynamic light scattering (DLS), and zeta potentials were used to characterize the SERS probes. The results of the characterization of the SERS probes are given in Figure 1. As can be seen from Figure 1A, superior SERS signals of ROX were obtained with hairpin-structured aptamer-modified Au NPs, and almost no obvious SERS signal of ROX was obtained using normal aptamer-modified Au NPs. We speculated that, with the hairpin-structured aptamer-modified Au NPs, the distance between the ROX and the Au NPs was suitable to achieve SERS enhancement, while for the normal aptamer-modified Au NPs, the distance between ROX and the Au NPs was too great to achieve SERS enhancement. The CD spectra of aptamers before and after forming hairpin structure were recorded to confirm the conformational change (Figure S2). As given in Figure S2, the intensities of both the negative peak and the positive peak in the CD spectra changed following heat and cold treatments, suggesting that the structure of the aptamers transformed. In addition, the positive peak and the negative peak at 240 nm and 260 nm in the CD spectrum of the treated aptamer indicated that the aptamer exhibited a B-form hairpin structure [37]. Additionally, the SERS spectra of the SERS probes, aptamers and 4-MBN-modified Au NPs are given in Figure 1B. As can be seen from Figure 1B, typical signals of ROX and 4-MBN can be clearly observed and distinguished in the SERS spectrum of the SERS probes. The characteristic SERS signals of ROX at Raman shifts of 1503 cm^−1^ and 1646 cm^−1^ can be attributed to the ring C-C stretching vibrations, and the characteristic peaks of 4-MBN at Raman shifts of 1074 cm^−1^ and 1586 cm^−1^ can be assigned to the C-S stretching and aromatic ring vibration, respectively [37,38].

The dynamic light scattering (DLS) results indicated that the main diameter of Au NPs was 51 nm, and the main diameter of the SERS probes increased to 66 nm (Figure 1C). This is probably due to the modification of the aptamers on the surface of the Au NPs. In addition, the morphology of the Au NPs was characterized by TEM. As can be observed in Figure 1C (inset panel), the synthesized Au NPs were uniform and oval in shape. Additionally, the results of the zeta potential measurements are given in Figure 1D. The results indicate that the SERS probes exhibited a greater negative zeta potential than the Au NPs. This can possibly be attributed to the extra negative charge brought by the phosphate skeleton in the aptamers [37]. In summary, these characterization results provide solid proof for the successful synthesis of the SERS probes.

### 2.3. Feasibility of Detecting Enrofloxacin Using the Ratiometric SERS Aptasensor

**Figure 2:**
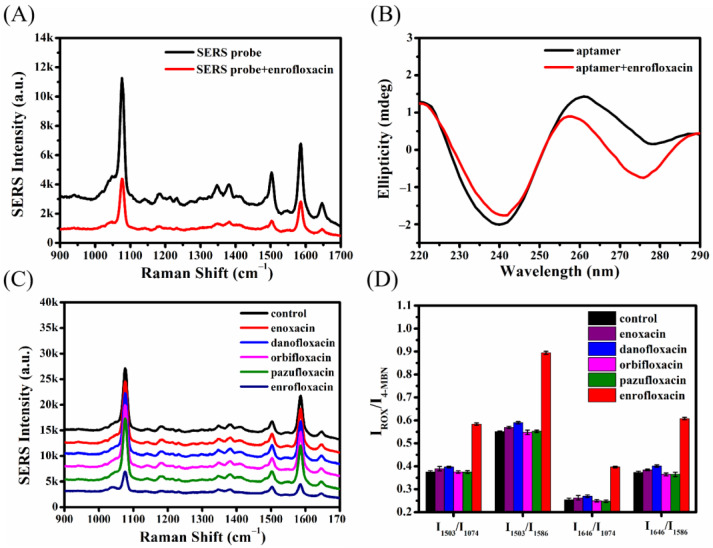
Results of the feasibility of detecting enrofloxacin using the proposed ratiometric SERS aptasensor. (**A**) SERS spectra of the probes before and after binding with enrofloxacin. (**B**) CD spectra of aptamer in the absence and presence of enrofloxacin. (**C**) SERS spectra of the probes incubated with enoxacin, danofloxacin, orbifloxacin, pazufloxacin and enrofloxacin at the concentration of 1 µM. (**D**) Ratios of the SERS signal intensities of ROX (I_ROX_) to 4-MBN (I_4-MBN_).

The SERS spectra of the probes before and after binding with enrofloxacin were collected, as shown in Figure 2A. It can be seen in Figure 2A that the intensities of the characteristic SERS signals of ROX decreased significantly after the probes were incubated with enrofloxacin. This result indicates that the presence of enrofloxacin causes a decrease in the intensities of the characteristic SERS signals of ROX. We speculated that when the aptamers in the SERS probes bound with enrofloxacin, their conformation would change and the ROX at their 3′ end would be moved far away from Au NPs, leading to a decrease in SERS signal intensities. The CD spectra of the aptamers in the absence and presence of enrofloxacin were recorded, and are presented in Figure 2B. As can be seen from Figure 2B, in the presence of enrofloxacin, the intensities of both the negative and positive peaks in the CD spectrum decreased, suggesting that the conformation change of the aptamers was induced by enrofloxacin.

Additionally, the specificity of the proposed ratiometric SERS aptasensor was investigated by comparing the SERS spectra of probes incubated with enoxacin, danofloxacin, orbifloxacin, pazufloxacin, and enrofloxacin at a concentration of 1 µM (Figure 2C). Enoxacin, danofloxacin, orbifloxacin, and pazufloxacin were selected as interference substances because their structures are similar to that of enrofloxacin. As can be seen from Figure 2C, only enrofloxacin was able to induce a decrease in SERS signal intensities. In addition, the ratios of the SERS signal intensities of ROX to 4-MBN are given in Figure 2D. It can be seen from Figure 2D that the ratios of the SERS signal intensities of ROX to 4-MBN dramatically increased in the presence of enrofloxacin, while no significant change in comparison to the control was observed in the presence of other antibiotics, indicating that the proposed ratiometric SERS aptasensor responds to enrofloxacin specifically.

### 2.4. Optimization of Experimental Parameters

#### 2.4.1. Optimization of Aptamer Concentration

**Figure 3:**
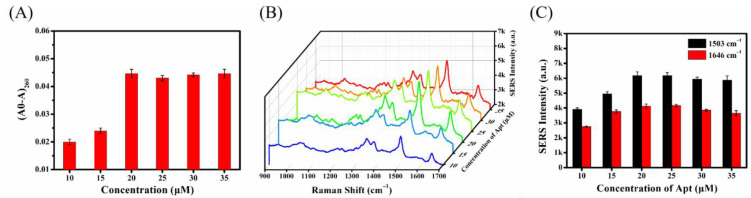
Results of optimizing the concentration of aptamer used to prepare the SERS probe. (**A**) Variations in UV-Vis absorption values of aptamer solutions at concentrations of 10–35 μM before (A0) and after (A) incubation with Au NPs. (**B**) SERS spectra of Au NPs modified with aptamers at concentrations of 10–35 μM. (**C**) Intensities of the characteristic SERS peaks of ROX at Raman shifts of 1503 cm^−1^ and 1646 cm^−1^.

The concentration of aptamers used to prepare the SERS probes directly affects the intensities of the SERS signals of ROX and the sensitivity of the ratiometric SERS aptasensor. Solutions of aptamers at concentrations of 10, 15, 20, 25, 30, 35 µM were used to prepare the SERS probes. The UV-Vis absorption values of the aptamer solutions before and after incubation with Au NPs and the SERS spectra of the corresponding SERS probes were collected to determine the optimal concentration of aptamer (Figure 3). The variations in the UV-Vis absorption values of the aptamer solutions before and after incubation with Au NPs are given in Figure 3A. It can be seen from Figure 3A that sufficient aptamers were modified on the surface of Au NPs when 20 µM aptamer solution was used to prepare the SERS probes, since the variations in UV-Vis absorption values exhibit no significant difference when the concentrations of aptamers are above 20 µM. Furthermore, the SERS spectra of probes prepared with different concentrations of aptamers are shown in Figure 3B. As can be observed in Figure 3B,C, the intensities of the SERS signals of ROX at Raman shifts of 1503 cm^−1^ and 1646 cm^−1^ increased significantly with increasing aptamer concentration in the range of 10–20 µM (*p* < 0.05). In addition, the SERS intensities reached a plateau when the concentrations of aptamer solutions were above 20 µM. Therefore, aptamer solution at a concentration of 20 µM was used to prepare the SERS probes.

#### 2.4.2. Optimization of 4-MBN Concentration

**Figure 4:**
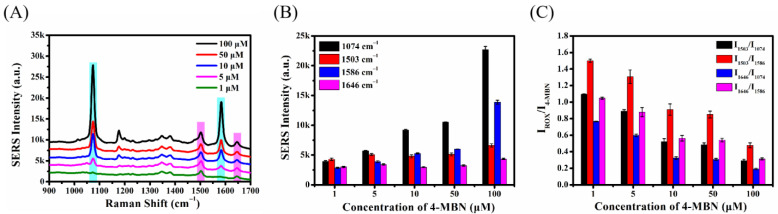
Results of optimizing the concentration of 4-MBN used to prepare the SERS probe. (**A**) SERS spectra of probes prepared with 4-MBN at the concentration of 1–100 μM. (**B**) Intensities of the characteristic SERS signals of ROX at Raman shifts of 1503 cm^−1^ and 1646 cm^−1^ and 4-MBN at Raman shifts of 1074 cm^−1^ and 1586 cm^−1^. (**C**) Ratios of the SERS signal intensities of ROX to 4-MBN.

The SERS signals of 4-MBN served as internal standards to correct interference in this study. The SERS spectra of probes prepared with 4-MBN solutions at concentrations of 1, 5, 10, 50, 100 µM were collected, as shown in Figure 4A. As can be seen from Figure 4A, the characteristic signals of ROX at Raman shifts of 1503 cm^−1^ and 1646 cm^−1^ and 4-MBN at Raman shifts of 1074 cm^−1^ and 1586 cm^−1^ can be clearly distinguished. In addition, comparisons of the intensities of the characteristic signals of ROX and 4-MBN and their ratios are given in Figure 4B,C. As shown in Figure 4B, the intensities of the SERS signals of 4-MBN increased gradually with increasing 4-MBN concentration, while intensities of the SERS signals of ROX showed no significant change. Thus, the ratios of the SERS intensities of ROX to 4-MBN decreased with increasing 4-MBN concentrations (*p* < 0.05). In addition, comparable intensities of SERS signals of ROX and 4-MBN were observed when 4-MBN solution at a concentration of 10 µM was used to prepare the SERS probes. Therefore, 10 µM was selected as the optimal concentration of 4-MBN. In addition, the ratios of the intensities of SERS signals of ROX at a Raman shift of 1503 cm^−1^ to that of 4-MBN at 1586 cm^−1^ were higher than the ratios of other pairs in all of the tested samples. Therefore, the SERS signals of ROX and 4-MBN at Raman shifts of 1503 cm^−1^ and 1586 cm^−1^ were selected as the optimal signals to calculate the ratio in the following analysis.

#### 2.4.3. Optimization of the Volume Ratio of SERS Probe to Enrofloxacin

**Figure 5:**
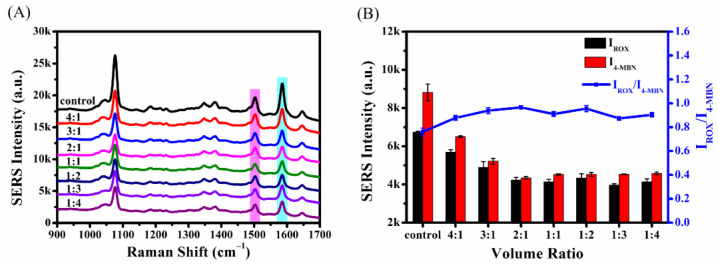
Results of optimizing the volume ratio of SERS probe to enrofloxacin. (**A**) SERS spectra of samples prepared with different volume ratios of the SERS probe to enrofloxacin (control: SERS probe). (**B**) Comparison of intensities of the characteristic SERS signals of ROX and 4-MBN at Raman shifts of 1503 cm^−1^ and 1586 cm^−1^ and their corresponding ratios.

The volume ratio of SERS probe to enrofloxacin was optimized in order to achieve excellent sensitivity. The SERS spectra of samples prepared with SERS probe and enrofloxacin at volume ratios of 4:1, 3:1, 2:1, 1:1, 1:2, 1:3, 1:4 and a constant total volume of 90 µL were collected and are given in Figure 5A. The intensities of the SERS signals of ROX and 4-MBN at Raman shifts of 1503 cm^−1^ (I_ROX_) and 1586 cm^−1^ (I_4-MBN_) and their corresponding ratios were compared, as shown in Figure 5B. The ratio of I_ROX_ to I_4-MBN_ first increased (*p* < 0.05) and then began to fluctuate slightly with decreasing volume ratios of SERS probe to enrofloxacin, and the largest value of I_ROX_ to I_4-MBN_ was observed at a volume ratio of 2:1. This is probably because adding too much enrofloxacin led to the dilution of the SERS probes in the detection system. Thus, the volume ratio of SERS probe to enrofloxacin was set as 2:1 in the following experiments.

#### 2.4.4. Optimization of Incubation Time

**Figure 6:**
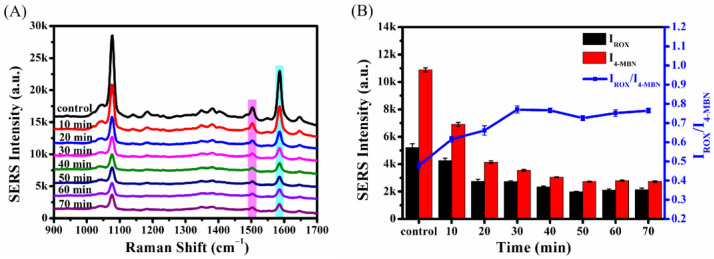
Results of optimizing the incubation time of SERS probes and enrofloxacin. (**A**) SERS spectra of samples prepared by incubating SERS probes and enrofloxacin for 10–70 min (control: SERS probe). (**B**) Intensities of SERS signals of ROX and 4-MBN at Raman shifts of 1503 cm^−1^ and 1586 cm^−1^ and their corresponding ratios.

The incubation time affects the capture efficiency of the SERS probe and the accuracy of the ratiometric SERS aptasensor. To select the optimal incubation time, SERS probes were incubated with 1 µM enrofloxacin for 10, 20, 30, 40, 50, 60, and 70 min, respectively. Figure 6A shows the SERS spectra collected from samples prepared with different incubation times. As can be seen from Figure 6A, the proposed ratiometric SERS aptasensor responded to enrofloxacin in 10 min, and the response values increased with increasing incubation time. The intensities of SERS signals of ROX and 4-MBN at Raman shifts of 1503 cm^−1^ and 1586 cm^−1^ and their corresponding ratios are given in Figure 6B. It can be seen in Figure 6B that the ratio of I_ROX_ to I_4-MBN_ first increased with increasing incubation time (*p* < 0.05) and then reached a plateau, with the largest ratio being observed at 30 min. Therefore, in the following experiments, SERS probes and enrofloxacin were incubated for 30 min before being analyzed by SERS.

#### 2.4.5. Optimization of Incubation Temperature

**Figure 7:**
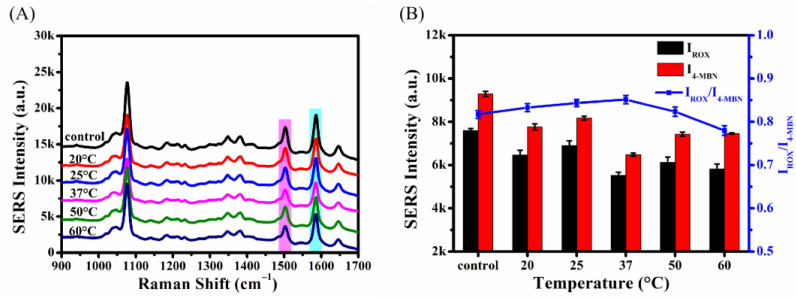
The results of optimizing the incubation temperature of SERS probes and enrofloxacin. (**A**) SERS spectra of samples prepared by incubating SERS probes and enrofloxacin at different temperatures (control: SERS probes incubated with 0.5× MOPS buffer at 25 °C). (**B**) Intensities of SERS signals of ROX at a Raman shift of 1503 cm^−1^ and 4-MBN at a Raman shift of 1586 cm^−1^ and their corresponding ratios.

Incubation temperature was optimized, since the capture of enrofloxacin by SERS probes is sensitive to temperature. The SERS spectra of probes incubated with 1 µM enrofloxacin at different temperatures (20, 25, 37, 50, 60 °C) were collected under the optimized experimental conditions and are given in Figure 7A. The control spectrum in Figure 7A was collected with SERS probes incubated with 0.5× MOPS buffer at 25 °C, since there were no significant differences among the spectra obtained using SERS probes incubated with 0.5× MOPS buffer at different temperatures. The SERS intensities of ROX at a Raman shift of 1503 cm^−1^ and 4-MBN at a Raman shift of 1586 cm^−1^ and their corresponding ratios are shown in Figure 7B. As can be seen from Figure 7B, at 37 °C, the ratio of I_ROX_ to I_4-MBN_ was significantly increased in comparison with the control (*p* < 0.05). Hence, 37 °C was chosen as the optimum incubation temperature.

### 2.5. Detection of Enrofloxacin

**Figure 8:**
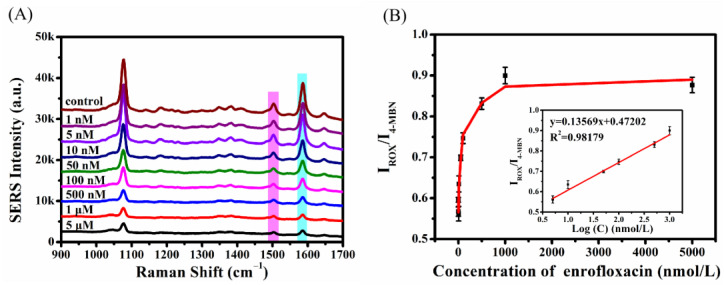
Determination of enrofloxacin using the established ratiometric SERS aptasensor. (**A**) SERS spectra of enrofloxacin at concentrations of 0 (control), 1 nM, 5 nM, 10 nM, 50 nM, 100 nM, 500 nM, 1 µM, and 5 µM. (**B**) Calibration curve (main panel) and linear dynamic range (inset panel) of the established ratiometric SERS aptasensor.

The SERS spectra of probes incubated with enrofloxacin at concentrations of 0 (control), 1 nM, 5 nM, 10 nM, 50 nM, 100 nM, 500 nM, 1 µM, and 5 µM under optimal conditions were collected and are shown in Figure 8A. It can be seen from Figure 8A that the intensities of the SERS spectra decrease with increasing enroflaxacin concentration. The ratios of I_ROX_ to I_4-MBN_ are plotted in Figure 8B. As can be seen from Figure 8B, the ratios of I_ROX_ to I_4-MBN_ increased with increasing enroflaxacin concentration, and then reached a plateau. In addition, the ratios of I_ROX_ to I_4-MBN_ exhibit a good linear relationship with logarithmic values at enrofloxacin concentrations from 5 nM to 1 µM, with a linear regression equation of y = 0.13569 x + 0.47202 and a correlation coefficient (R^2^) of 0.98. The limit of detection (LOD) was calculated using the equation of LOD = 3SD/k, in which SD was the standard deviation of the blank sample, and k was the slope of the calibration curve. The calculated LOD was 0.12 nM (0.043 ppb).

In addition, the established ratiometric SERS aptasensor was compared with previously reported methods for the detection of enrofloxacin (Table 1). Table 1 shows that the established ratiometric SERS aptasensor exhibits a relatively wide linear range and low LOD in comparison with other methods for the detection of enrofloxacin. Additionally, the established ratiometric SERS aptasensor is easy to use and rapid to respond. More importantly, the established ratiometric SERS aptasensor responded to enrofloxacin with great specificity due to the unique recognition of the target by the aptamer. In addition, the whole process could be completed within 40 min. Therefore, the established ratiometric SERS aptasensor shows potential for application as a reliable and rapid detection technique for the determination and quantification of enrofloxacin.

### 2.6. Reproducibility, Stability and Uniformity of the Established Ratiometric SERS Aptasensor

**Figure 9:**
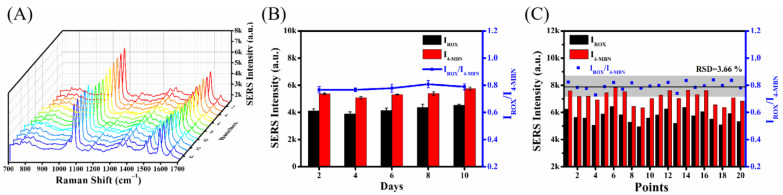
Evaluation of the established ratiometric SERS aptasensor. (**A**) Reproducibility. (**B**) Stability. (**C**) Uniformity.

Reproducibility was investigated by comparing the SERS spectra collected from three independent batches of enrofloxacin samples at a concentration of 1 µM. As can be seen from Figure 9A, no significant differences were observed in the SERS spectra of different samples, which suggested that the results obtained using the established ratiometric SERS aptasensor for enrofloxacin detection were highly reproducible. Moreover, the stability of the sensor was investigated by comparing the SERS spectra collected with probes stored for 2–10 days at 4 °C. Figure 9B shows that the intensities of the SERS signals of ROX at a Raman shift of 1503 cm^−1^ and 4-MBN at a Raman shift of 1586 cm^−1^, as well as their corresponding ratios, exhibited no obvious difference, and the RSD of ratio of I_ROX_ to I_4-MBN_ was 2.25%, indicating that the established ratiometric SERS aptasensor presented excellent stability. Additionally, the uniformity of the sensor was evaluated by comparing 20 spectra collected from different points of the same sample. As can be seen from Figure 9C, the ratios of I_ROX_ to I_4-MBN_ were highly consistent, with an RSD of 3.66%. Therefore, the above results demonstrate that the established ratiometric SERS aptasensor exhibited excellent performance in the determination of enrofloxacin.

### 2.7. Application in the Determination of Enrofloxacin in Real Samples

The performance of the established ratiometric SERS aptasensor in real sample analysis was evaluated by detecting enrofloxacin in fish and chicken meat. The samples were first spiked with enrofloxacin, with final concentrations of 10, 100, and 500 nM, and then the enrofloxacin concentrations of the blank and spiked samples were analyzed using the developed ratiometric SERS aptasensor. The SERS spectra for the detection of enrofloxacin in fish and chicken meat are given in Figures S3 and S4, respectively. The results are summarized in Table 2. As can be observed in Table 2, the recoveries for the determination of enrofloxacin in fish and chicken meat were in the range of 93.6–112.0%, with RSDs ranging from 0.7% to 2.7% (*n* = 3). These results indicate that the ratiometric SERS aptasensor exhibited high accuracy and good reliability for the detection of enrofloxacin in meat.

## 3. Materials and Methods

### 3.1. Materials

Chloroauric acid tetrahydrate (HAuCl_4_·4H_2_O, 47.8%), and 3-(N-Morpholino)-propane sulfonic acid sodium (MOPS, 99.5%) were supplied by Yuanye Bio-Technology Co., Ltd. (Shanghai, China). Tris-(2-carboxyethyl) phosphine hydrochloride (TCEP), trisodium citrate dihydrate (C_6_H_5_Na_3_O_7_⋅2H_2_O, 99.0%), acetonitrile, *n*-butanol, 4-mercaptobenzonitrile (4-MBN), enoxacin (≥98%), danofloxacin (≥98%), orbifloxacin (≥99%), pazufloxacin (≥98%) and enrofloxacin (≥98%) were obtained from Aladdin Co., Ltd. (Shanghai, China). Sodium hydroxide (NaOH, ≥96%), sodium chloride (NaCl), and 5× TBE buffer were purchased from Sinopharm Chemical Reagent Co., Ltd. (Shanghai, China). All reagents were of analytical grade and were used without further purification unless otherwise mentioned. Distilled water was used in all experiments.

In reference to the sequence of a truncated aptamer for enrofloxacin reported by Sha et al. [21], the sequence of the aptamer was designed as 5′-HS-SH-(CH_2_)_6_-TTTGGGTTATTTCAGGGGGACCCATCAGGGGGCTAGGCTAACCC-ROX-3′ in this study in order to obtain good SERS signals. The designed aptamer was synthesized and purified by Sangon Biotechnology Co., Ltd. (Shanghai, China).

### 3.2. Synthesis of Au NPs

Au NPs were prepared according to a previously published sodium citrate reduction method with minor adjustment [24]. Briefly, 30 mL of distilled water and 300 µL of 1% (*w/v*) HAuCl_4_·4H_2_O were added into a 100 mL clean conical flask. The mixture was stirred by a magnetic stirrer at a speed of 250 rpm and rapidly heated to boil. As soon as the solution was boiling, 180 µL of 1% (*w/v*) sodium citrate tribasic dihydrate solution was added into the solution. After boiling for about 5 min, when the color of the colloid turned to cloudy claret, the conical flask was removed from heat and the synthesized Au NPs colloid was cooled to room temperature. After cooling, the synthesized Au NPs colloid was stored at 4 °C for further use. The diameter of the prepared Au NPs was analyzed using a Litesizer 500 Zeta potentiometer (Anton Paar, Graz, Austria) using 3 mL at 1:50 (Stock:H_2_O) dilution. TEM images were collected using a FEI Tecnai G20 (FEI Company, Hillsboro, OR, USA) with 1:5 (Stock:H_2_O) dilution dried on carbon-coated copper grid.

### 3.3. Preparation of the SERS Probe

The SERS probe was prepared using a rapid and efficient instant dehydration in butanol (INDEBT) method reported by Yan Hao et al. [45] with minor modifications. Briefly, 8 µL of 100 µM thiolated aptamer was annealed at 95 °C for 10 min and then immediately cooled on ice for 20 min to form a stable hairpin structure. Then, 10 µL of 100 mM TCEP was added into the hairpin aptamer solution and incubated for 30 min to cleave the disulfide bond. Next, 85 µL homemade Au NPs colloids was mixed with 1 µL of 20 µM aptamer solution and incubated for 20 min at room temperature. Subsequently, 4 µL of 150 mM NaCl and 10 µL of 10 µM 4-MBN were added to the mixture. After 5 min, the mixture was mixed with 900 μL n-butanol and vortex-mixed for several seconds. After that, 200 μL of 0.5× TBE (pH 8.0) buffer was added to the above solution and vortex-mixed for several seconds to accelerate the separation of liquid phases. Finally, the solution was centrifuged at 6500 rpm for 5 min and the sediments were washed with distilled water three times to remove the excess aptamer and 4-MBN, the obtained SERS probes were stored at 4 °C until further use. Circular dichroism (CD) was used to characterize the conformation of aptamer. CD spectra of aptamer solutions treated with and without 95 °C heating, and before and after binding with enrofloxacin were recorded using a circular dichroism spectrometer (Applied Photophysics, London, UK). All tests were conducted in triplicate in the range of 220–290 nm at a scan rate of 100 nm/min, and the average CD spectrum was used to represent the corresponding sample.

### 3.4. SERS Detection of Enrofloxacin

Firstly, the prepared SERS probes were resuspended in 0.5× MOPS buffer solution. Then, enrofloxacin standard solutions at different concentrations were prepared with 0.5× MOPS buffer, and 30 µL of each enrofloxacin solution was mixed with 60 µL of SERS probe, unless otherwise stated. Subsequently, the mixture was incubated for 30 min at 37 °C to ensure thorough capture of enrofloxacin by the SERS probes. After incubation, the mixture was centrifugated at 6500 rpm for 5 min and the precipitated SERS probes were collected and resuspended in 10 µL deionized water. After that, the suspension was dropped on a tin-foil-wrapped glass slide and dried at room temperature. After drying, the sample spots were analyzed using a portable Raman spectrometer (i-Raman Plus BWS465-785H spectrometer, B&W Tek, Newark, DE, USA) with a 785 nm laser and an acquisition time of 10 s and an accumulation time of 3 s. Five representative SERS spectra were collected for each sample.

### 3.5. Detection of Enrofloxacin in Meat Samples

Fish and chicken meat purchased from a local supermarket was artificially contaminated with enrofloxacin, and then detection was performed using the established ratiometric SERS aptasensor. Before detection, samples were pre-treated according to a National Food Safety Standard method of China (GB 31656. 3-2021) with minor modifications. Briefly, the meat samples were first cut into pieces and homogenized using a blender. Then, 5 g of the blended meat samples was mixed with 20 mL acidified acetonitrile and 1 mL of enrofloxacin standard solution to achieve final concentrations of 10 nM, 100 nM, and 500 nM, respectively. Subsequently, the mixture was vortex mixed for 2 min and centrifuged at 3000 rpm for 5 min, and the obtained supernatant was diluted 10 times using 0.5× MOPS buffer in order to reduce the concentration of acidified acetonitrile and to create the optimum conditions for the recognition of enrofloxacin using SERS probes. Finally, the pH was adjusted to 7 with 2 M NaOH, then subjected to SERS detection.

## 4. Conclusions

In summary, a reliable, rapid, and sensitive ratiometric SERS aptasensor was developed using ROX-labeled aptamers and 4-MBN-modified Au NPs as the SERS probe for the detection and quantification of enrofloxacin in this study. In the presence of enrofloxacin, the conformation of the aptamers transformed due to the specific recognition of enrofloxacin by the aptamers, leading to an increase in the distance between ROX and Au NP and a decrease in the intensities of SERS signals of ROX. Meanwhile, the intensity of the SERS signal of 4-MBN at a Raman shift of 1586 cm^−1^ was used as an internal standard to remove interference. Accurate and sensitive determination of enrofloxacin was realized using the ratio of the SERS signal intensities of ROX to 4-MBN. Under optimal conditions, the developed ratiometric SERS aptasensor displayed a good linear relationship between the ratios of the SERS signal intensities of ROX to 4-MBN and the logarithmic values of the concentrations of enrofloxacin in the range of 5 nM to 1 µM, with an R^2^ of 0.98 and an LOD of 0.12 nM (0.043 ppb). Furthermore, the developed ratiometric SERS aptasensor was successfully applied to the determination of enrofloxacin in fish and chicken meat, with recovery values of 93.6–112.0%. Additionally, the established ratiometric SERS aptasensor exhibited excellent reproducibility, stability, and uniformity. Therefore, the established ratiometric SERS aptasensor provided a new strategy for the rapid detection and quantification of enrofloxacin, and had the potential to be applied for the rapid and on-site determination of enrofloxacin in complex matrices.

## Figures and Tables

**Table 1:** Comparison of the established ratiometric SERS aptasensor with previously reported methods for enrofloxacin detection.

Analytical Method	Linear Range (M)	R^2^	LOD (M)	Ref.
Localized surface plasmon resonance (LSPR)	6.95 × 10^−8^–2.78 × 10^−6^	0.98	1.70 × 10^−9^	[39]
Molecular imprinting technique (MIT)	2.70 × 10^−10^–2.70 × 10^−8^	0.996	1.39 × 10^−10^	[40]
Colorimetry	5.0 × 10^−9^–1.0 × 10^−7^	0.99702	1.89 × 10^−9^	[21]
Fluorescence	2.70 × 10^−9^–1.70 × 10^−7^	0.98	1.30 × 10^−9^	[41]
Photoelectrochemical (PEC)	2.0 × 10^−12^–1.0 × 10^−7^	0.994	8.30 × 10^−10^	[42]
Electrochemical immunosensor (ECI)	1.39 × 10^−8^–2.78 × 10^−8^	0.975	8.30 × 10^−9^	[10]
SERS	2.78 × 10^−5^–1.39 × 10^−3^	0.9976	1.98 × 10^−6^	[29]
SERS	2.80 × 10^−10^–2.80 × 10^−5^	0.97	1.0 × 10^−10^	[43]
SERS	1.0 × 10^−7^–1.0 × 10^−3^	0.99	1.0 × 10^−7^	[44]
Ratiometric SERS aptasensor	5.0 × 10^−9^–1.0 × 10^−6^	0.98	1.20 × 10^−10^	This work

**Table 2:** Results of detection of enrofloxacin in fish and chicken meat using the developed ratiometric SERS aptasensor.

Sample	Spiked Concentration (nM)	Detected Concentration (nM)	Recovery (%)	RSD (%) (*n* = 3)
Fish	-	-		
	10.0	9.4 ± 0.9	94.2	1.2
	100.0	112.0 ± 7.9	112.0	1.0
	500.0	424.5 ± 46.4	93.6	2.7
Chicken	-	-		
	10.0	9.5 ± 0.8	95.3	0.7
	100.0	102.9 ± 16.9	102.9	2.1
	500.0	517.3 ± 35.6	103.4	2.6

-: not added/detected.

## Data Availability

The data presented in this study are available in article or Supplementary Materials.

## Supplementary Materials

The following supporting information can be downloaded at: https://www.mdpi.com/article/10.3390/molecules27248764/s1, Figure S1: The secondary structure of the designed aptamer; Figure S2: CD spectra of aptamers before and after forming hairpin structure; Figure S3: Results of detecting enrofloxacin in fish meat at the concentrations of 10, 100, and 500 nM. Figure S4: Results of detecting enrofloxacin in chicken meat at the concentrations of 10, 100, and 500 nM.
