# Supplementary material for: Reliable and Rapid Detection and Quantification of Enrofloxacin Using a Ratiometric SERS Aptasensor
Source: Molecules. 2022 Dec 10;27(24):8764. doi: 10.3390/molecules27248764 (PMC9784490; doi:10.3390/molecules27248764)
Supplement: Supplementary file 1 [file molecules-27-08764-s001.zip › molecules-2093288-supplementary.pdf]

Supplementary Materials for:

Reliable and Rapid Detection and Quantification of Enrofloxacin Using a Ratiometric SERS Aptasensor

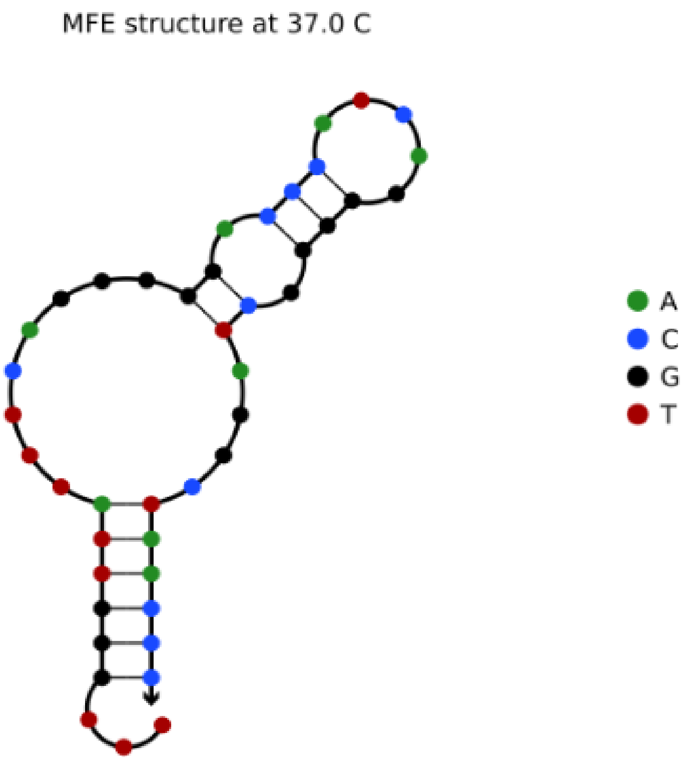

Figure S1. Secondary structure of the aptamer.

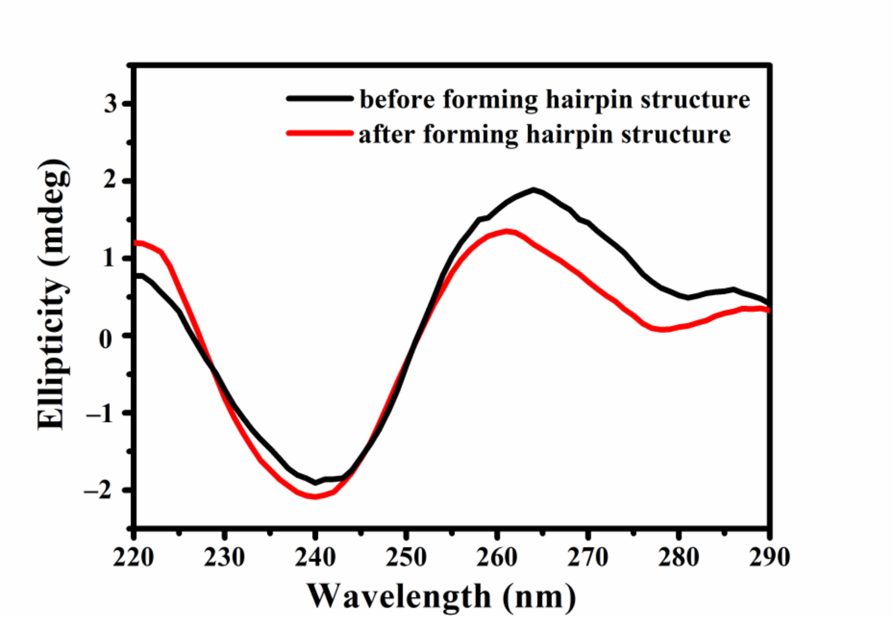

Figure S2. CD spectra of aptamers before and after forming hairpin structure.

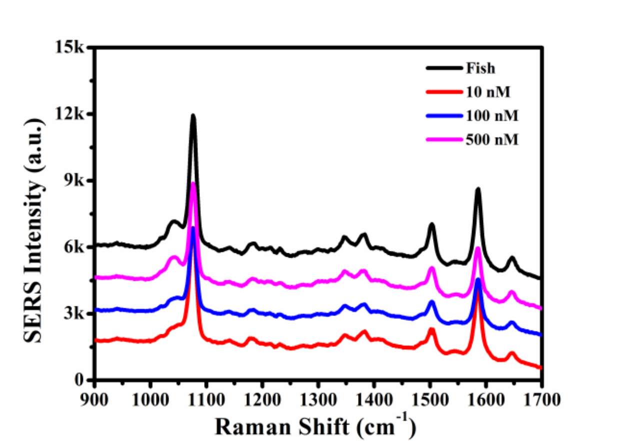

Figure S3. Results of detecting enrofloxacin in fish meat at the concentrations of 10, 100, and 500 nM.

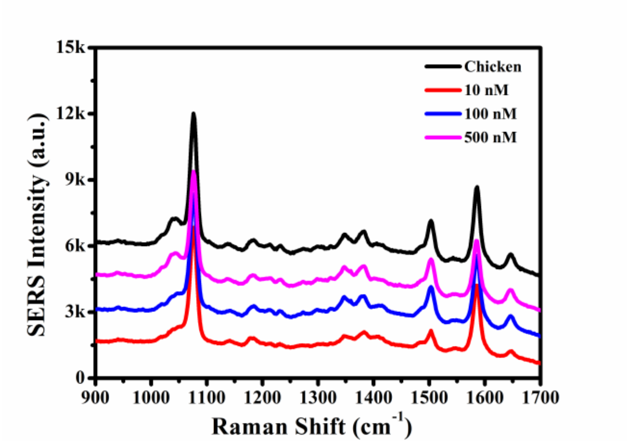

Figure S4. Results of detecting enrofloxacin in chicken meat at the concentrations of 10, 100, and 500 nM.
